# Very low-calorie ketogenic diet (VLCKD): a therapeutic nutritional tool for acne?

**DOI:** 10.1186/s12967-024-05119-5

**Published:** 2024-03-31

**Authors:** Ludovica Verde, Evelyn Frias-Toral, Sara Cacciapuoti, Daniel Simancas-Racines, Matteo Megna, Giuseppina Caiazzo, Luca Potestio, Maria Maisto, Gian Carlo Tenore, Annamaria Colao, Silvia Savastano, Giovanna Muscogiuri, Luigi Barrea

**Affiliations:** 1 Department of Public Health, University of Naples Federico II, Via Sergio Pansini 5, 80131 Naples, Italy; 2 Centro Italiano per la cura e il Benessere del Paziente con Obesità (C.I.B.O), Unità di Endocrinologia, Diabetologia e Andrologia, Dipartimento di Medicina Clinica e Chirurgia, Università degli Studi di Napoli Federico II, Via Sergio Pansini 5, 80131 Naples, Italy; 3 School of Medicine, Universidad Espíritu Santo, Samborondón, 0901952 Ecuador; 4 Section of Dermatology-Department of Clinical Medicine and Surgery, University of Naples Federico II, Naples, Italy; 5 Centro de Investigación en Salud Pública y Epidemiología Clínica (CISPEC), Facultad de Ciencias de la Salud Eugenio Espejo, Universidad UTE, Quito, 170129 Ecuador; 6 Dipartimento di Scienze Biomediche avanzate, Università Degli Studi Di Napoli Federico II, Naples, Italy; 7 Department of Pharmacy, University of Naples “Federico II”, Naples, Italy; 8 Unità di Endocrinologia, Diabetologia e Andrologia, Dipartimento di Medicina Clinica e Chirurgia, Università degli Studi di Napoli Federico II, Via Sergio Pansini 5, 80131 Naples, Italy; 9 Cattedra Unesco “Educazione Alla Salute E Allo Sviluppo Sostenibile”, University Federico II, Naples, Italy; 10 Dipartimento di Benessere, Nutrizione e Sport, Università Telematica Pegaso, Centro Direzionale, Via Porzio, Isola F2, 80143 Naples, Italy

**Keywords:** Acne, Very low-calorie ketogenic diet, VLCKD, Ketogenic diet, Obesity, Inflammation, Oxidative stress, Diet, Nutrition

## Abstract

**Background:**

Acne, a chronic inflammatory disease impacting the pilosebaceous unit, is influenced significantly by inflammation and oxidative stress, and is commonly associated with obesity. Similarly, obesity is also associated with increased inflammation and oxidation. The role of diet in acne remains inconclusive, but the very low-calorie ketogenic diet (VLCKD), known for weight loss and generating anti-inflammatory ketone bodies, presents promising potential. Despite this, the effects of VLCKD on acne remain underexplored. This study aimed to investigate the efficacy of a 45-day active phase of VLCKD in reducing the clinical severity of acne in young women with treatment-naïve moderate acne and grade I obesity.

**Methods:**

Thirty-one women with treatment-naïve moderate acne, grade I obesity (BMI 30.03–34.65 kg/m^2^), aged 18–30 years, meeting inclusion/exclusion criteria, and consenting to adhere to VLCKD were recruited. Baseline and post-intervention assessments included anthropometric measurements, body composition, phase angle (PhA), trimethylamine N-oxide (TMAO) levels, and reactive oxygen metabolite derivatives (dROMs) as markers of inflammation, dysbiosis, and oxidative stress, respectively. A comprehensive dermatological examination, incorporating the Global Acne Grading System (GAGS) and the Dermatology Life Quality Index (DLQI), was conducted for all women.

**Results:**

VLCKD resulted in general improvements in anthropometric and body composition parameters. Significantly, there were significant reductions in both the GAGS score (Δ%: − 31.46 ± 9.53, *p* < 0.001) and the DLQI score (Δ%: − 45.44 ± 24.02, *p* < 0.001) after the intervention. These improvements coincided with significant decreases in TMAO (*p* < 0.001) and dROMs (*p* < 0.001) levels and a significant increase in PhA (Δ%: + 8.60 ± 7.40, *p* < 0.001). Changes in the GAGS score positively correlated with changes in dROMs (*p* < 0.001) and negatively with PhA (*p* < 0.001) even after adjusting for Δ% FM. Changes in the DLQI score positively correlated with changes in dROMs (*p* < 0.001) and negatively with PhA (*p* < 0.001) even after adjustment for Δ% FM.

**Conclusion:**

Given the side effects of drugs used for acne, there is an increasing need for safe, tolerable, and low-cost treatments that can be used for acne disease. The 45-day active phase of VLCKD demonstrated notable improvements in acne severity, and these improvements seemed to be attributable to the known antioxidant and anti-inflammatory effects of VLCKD.

**Graphical Abstract:**

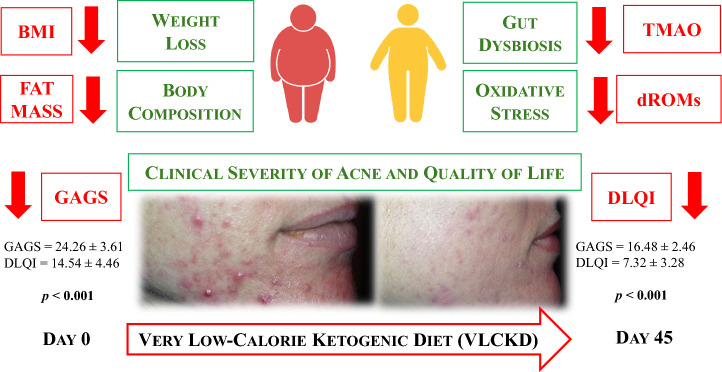

**Supplementary Information:**

The online version contains supplementary material available at 10.1186/s12967-024-05119-5.

## Introduction

Acne vulgaris (acne) is a complex, chronic inflammatory skin disease involving the pilosebaceous unit [1]. The prevalence of acne varies by time and country, and lifestyle may influence it [2, 3]. This skin condition affects 70–80% of adolescents and persists into the 20 s and 30 s in about 64% and 43% of affected individuals, respectively [4, 5]. In addition, several studies show that acne is more common in adult females when compared to adult males [6, 7]. Of interest, Chang J et al. reported a 1.5-fold higher proportion of dermatology visits for acne among women compared to men ages 20–29 years [8]. This sex difference in dermatological care may be tied to an increased acne severity among adult women as well as an increased impact on quality of life in this population [6–8]. In this context, acne patients may have significant quality of life (QoL) impairment [9] and its assessment, as an integral part of acne management in these patients, is recommended by several international guidelines [10, 11]. The Dermatology Life Quality Index (DLQI) is the most widely used health-related quality of life questionnaire in dermatology, particularly in studies on acne [10, 12].

Acne can result in enduring scarring and hyperpigmentation [13, 14], necessitating effective prevention and treatment to mitigate its significant impact on patients' quality of life [13]. A characteristic feature of acne patients is lesion pleomorphism, where different types of lesions, both inflammatory (such as papules, pustules, and nodules) and non-inflammatory (like comedones), may coexist in the same individual [1]. The clinical manifestations of acne can vary widely based on factors such as the severity, number, and type of predominant lesions [1].

The multifaceted pathogenesis of acne is attributed to several factors, including hyperseborrhea, hyperkeratinization of the pilosebaceous duct, colonization by *Propionibacterium acnes*, and perifollicular inflammation [15]. Abnormal desquamation of the sebaceous follicle epithelium (comedogenesis), sebaceous gland hyperplasia with seborrhea, increased bacterial colonization, and immunologic and inflammatory elements are the main pathophysiologic factors influencing acne development [15].

Key players in acne pathophysiology involve complex immunochemical pathways associated with inflammation, encompassing various inflammatory mediators and their target receptors, such as cytokines, defensins, peptidases, sebaceous lipids, and neuropeptides [16]. Elevated levels of prostaglandin E2 and peroxisome proliferator-activated receptor (PPAR)-γ can contribute to sebaceous gland hyperplasia and excessive sebum production, leading to inflammation and acne lesions [17]. *Propionibacterium acnes* also plays a role in triggering the release of pro-inflammatory cytokines [17].

In addition to increased sebum production and altered keratinization, recent discoveries highlight the microbiome as a third major player in acne development, interacting with the innate immune system [18]. The intestinal flora's influence on acne is speculated to involve interactions with the mammalian target of rapamycin (mTOR) pathway [19–21]. Metabolites from the gut microbiota may regulate cell expansion, fat metabolism, and metabolic functions through the mTOR pathway [22]. The interplay between mTOR and gut microbiota may form a mechanism by which the intestinal flora exacerbates acne, particularly in cases of gut dysbiosis and a disrupted intestinal barrier, creating a positive feedback loop and amplifying host metabolism and inflammation [23].

Apart from the traditional factors linked to acne, recent findings have established a connection between oxidative stress and the development of this condition [24]. Notably, strains associated with acne could release porphyrins, leading to an escalation in reactive oxygen species (ROS) formation and initiating an inflammatory response in keratinocytes [25]. This inflammatory process is linked to an imbalance between oxidants and antioxidants [24]. The role of ROS in acne vulgaris pathogenesis is significant, influencing the mTOR pathway, PPAR, toll-like receptor (TLR), and the innate immune system, thereby causing inflammation through alterations in the production of various pro-inflammatory cytokines like tumor necrosis factor (TNF)-α, interleukin (IL)-8, and IL-1 [24].

Obesity, a condition characterized by excess body weight and adipose tissue accumulation, has been associated with various inflammatory and metabolic disorders [26]. However, the link between obesity and acne is not fully understood, but emerging evidence suggests a potential connection through inflammatory and hormonal mechanisms [27]. Obesity is known to induce a state of chronic low-grade inflammation, marked by increased levels of pro-inflammatory cytokines and adipokines [26]. This inflammatory state may contribute to the development and exacerbation of acne by influencing the pathways involved in sebum production, follicular hyperkeratinization, and immune responses in the skin [27]. Additionally, obesity is often associated with insulin resistance and elevated levels of insulin-like growth factor 1 (IGF-1) [28], both of which have been implicated in the pathogenesis of acne [1]. Moreover, the gut microbiota, which plays a crucial role in maintaining overall health, can be altered in individuals with obesity [29, 30]. The interplay between obesity, gut dysbiosis, and acne may involve complex interactions among inflammation, hormonal regulation, and the immune system [31]. Very low-calorie ketogenic diet (VLCKD) has been shown to have anti-inflammatory and antioxidant effects, improve insulin sensitivity, and modulate the gut microbiota. Addressing acne through dietary interventions, such as VLCKD, could potentially impact both acne and the associated inflammatory and gut dysbiosis components [31].

To date, there is a large gap in the scientific literature on the use of VLCKDs for skin diseases. While there is some evidence supporting the use of ketogenic diets in psoriasis [32, 33], to our knowledge, no studies to date have evaluated the efficacy of VLCKD in reducing the clinical severity of acne. Thus, considering the existence of inflammation, oxidative stress, and dysbiosis in patients with acne, we suppose that a highly antioxidant and anti-inflammatory dietary therapy such as VLCKD, beyond the well-known weight loss effects, can contribute to improve both oxidation and dysbiosis and, consequently, improve the clinical severity of acne. In this context, the main aim of this study was to evaluate the efficacy of 45 days of active phase of VLCKD in reducing the clinical severity of acne in a group of young women with treatment-naïve moderate acne and grade I obesity.

## Materials and methods

### Population study

This study included 31 treatment-naïve women affected by moderate acne attending the outpatient clinic of the Units of Endocrinology and Dermatology of Federico II University Hospital. Ethical approval for the study was obtained from the Local Ethics Committee (reference no. 50/20), and all procedures adhered strictly to the World Medical Association's Code of Ethics, particularly the Declaration of Helsinki, outlining principles for human experimentation. The study's objectives and procedures were clearly communicated to all women, and written informed consent, expressing their willingness to participate, was obtained before their involvement.

At baseline, all women were assessed during the follicular phase of the menstrual cycle, and a comprehensive medical history, including drug usage, was documented. Inclusion criteria encompassed young women of childbearing age (18–30 years) with untreated moderate acne (Global Acne Grading System—GAGS—scores ranging from 19 to 30) and grade I obesity (BMI 30.0–34.9 kg/m^2^). To enhance sample homogeneity, only non-smoking women with no regular physical activity (less than 30 min of aerobic exercise *per* day) and meeting specific criteria were included, while those with certain exclusion criteria were omitted:

1. Age < 18 years and > 30 years;
2. Women with mild or severe acne;
3. Women with any other active skin condition (e.g., psoriasis or hidradenitis suppurativa) that might interfere with acne assessment;
4. Presence of one or more contraindications for VLCKD as *per* current European Association for the Study of Obesity (EASO) guidelines [34];
5. Women with a medical history affecting blood glucose or insulin concentrations, including diabetes types 1 and 2, prediabetes, or insulin resistance (Homeostatic model assessment for insulin resistance > 2.5), and/or taking medications altering blood glucose levels or insulin concentrations;
6. Women with acne lasting > 6 months or receiving systemic acne treatment for at least 3 months;
7. Pregnant or lactating women in the past 6 months;
8. Women with a self-reported recent weight change (> 10% weight change within the last 6 months);
9. Endocrine disorders affecting body composition or nutritional status, including biochemical hyperandrogenaemia and/or hyperandrogenism, oligomenorrhea due to polycystic ovarian syndrome, or secondary etiologies (according to the Endocrine Society) [35];
10. Chronic diseases affecting fluid homeostasis, such as liver or renal chronic diseases, cancer and acute or chronic inflammatory diseases;
11. Use of drugs impacting body composition, nutrient metabolism, or weight loss;
12. Dietary regimens in the last three months, including ketogenic diets, vegan or vegetarian diets, or supplementation with antioxidants, vitamins, or minerals;
13. Women with implanted pacemakers or defibrillators due to the theoretical risk of interference with the bioelectrical impedance analysis (BIA) device activity.

**Fig. 1:**
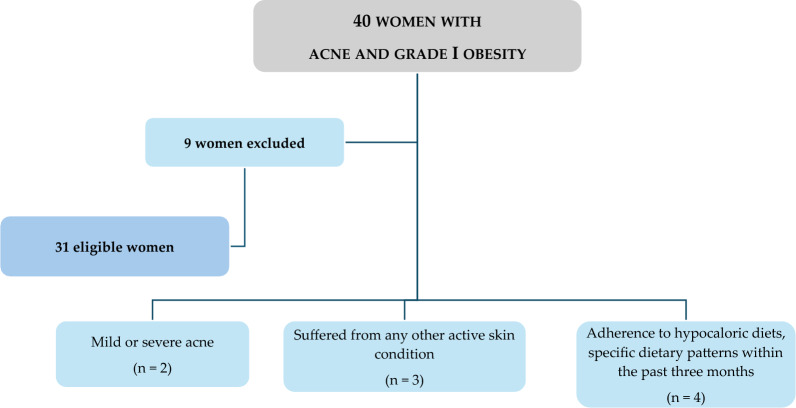
Flow chart of the study participants

Figure 1 shows a flow chart of included and excluded women.

### Study protocol

**Fig. 2:**
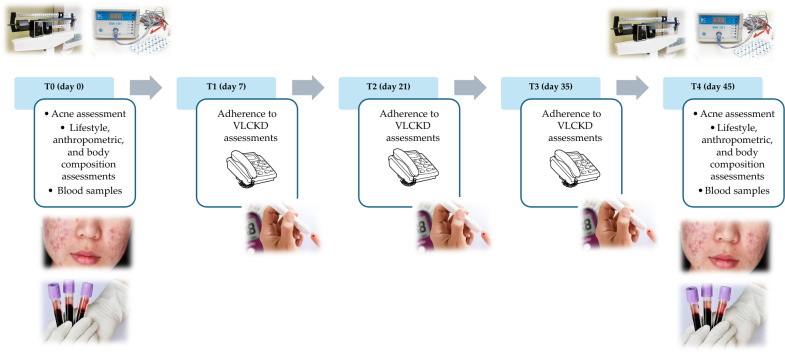
Study protocol

The study protocol encompassed a series of five visits (T0-day 0, T1-day 7, T2-day 21, T3-day 35, T4-day 45) over a total span of 45 days (Fig. 2). In detail, at baseline (T0), a comprehensive assessment carried out by a team of Endocrinologist, Dermatologist, and Nutritionist was conducted to ascertain the eligibility of patients. Those meeting the criteria for inclusion and exclusion were enrolled in the study and provided their written informed consent. At this point, the Endocrinologist carried out the first medical examination to ascertain the inclusion criteria for the study. Then, the dermatologist performed the clinical acne assessment and confirmed the inclusion criteria for each patient. Finally, the Nutritionist carried out nutritional status assessments (anthropometry and body composition) and drew up the VLCKD dietary therapy. All participants were then given personalized instructions for adhering to the diet. Simultaneously, with the support of nursing staff, blood samples were collected for general biochemical tests, oxidative stress evaluation, and trimethylamine N-oxide (TMAO) levels. Finally, women were advised to maintain the same lifestyle habits.

In the subsequent follow-up visits (T1-day 7, T2-day 21, and T3-day 35), a nutritionist carried out a telephone interview to evaluate adherence to the diet and the state of ketosis.

Adherence to the diet was assessed by asking the patient if she was consuming the number of VLCKD replacement meals, if she was drinking at least 2 L of water *per* day, and if she was respecting the written indications on dietary therapy. Ketosis status was assessed through ketone body measurements extracted from capillary blood samples, and the Nutritionist recorded only whether the patient had ketosis or not (YES/NO).

In all of these follow-up visits, the Nutritionist also documented any changes in physical activity levels or food and drink consumption patterns outlined in the VLCKD protocol.

In the last visit (T4-day 45), a final round of endocrinological, dermatological, and nutritional assessments was conducted. Blood samples were collected once more for the repetition of oxidative stress and TMAO analyses.

### Acne severity assessment

Every woman underwent a comprehensive dermatological examination, which included the use of the GAGS, a quantitative scoring system designed to evaluate the severity of acne. Initially developed by Doshi et al. [36], the GAGS score is calculated by adding six regional subscores. Specifically, each point is determined by multiplying the factor assigned to each region (3 for the chest and upper back, 1 for the chin and nose, and 2 for the forehead and each cheek) by the highest weighted lesion within that region (4 for ≥ one nodule, 3 for ≥ one pustule, 2 for ≥ one papule, and 1 for ≥ one comedone). These regional factors consider the density of pilosebaceous units, their surface area, and distribution. The cumulative local scores yield the global GAGS score, ranging from 0 to 52. The severity of acne was categorized into three GAGS groups: mild (GAGS score 1–18, characterized by several non-inflammatory comedones with fewer inflammatory lesions), moderate (GAGS score from 19 to 30, marked by numerous comedones, papules, and pustules, but without nodules), and severe (GAGS score from 31 to 38, indicating the presence of inflammatory nodules in addition to papules and pustules) [32, 33]. A single experienced dermatologist clinically assessed the GAGS score. To prevent rating biases, the dermatologists conducting the evaluations were kept unaware of the study's design [36, 37].

### Quality of life assessment

Women in the study filled out the DLQI, a questionnaire consisting of 10 items designed to evaluate the overall impact of skin disease on quality of life [12]. The total score spans from 0 to 30, where 0 signifies no influence of the skin disease on quality of life, and 30 indicates the maximum impact on quality of life. The grading system is as follows: 0–1 denotes no effect on the patient’s life, 2–5 signifies a small effect, 6–10 indicates a moderate effect, 11–20 suggests a very large effect, and 21–30 implies an extremely large impact on the woman’s life [12].

### Anthropometric measurements

Anthropometric assessments were conducted by a certified clinical nutrition specialist, adhering to the International Society for the Advancement of Kinanthropometry (ISAK 2006) guidelines. The measurements were performed in the morning, between 8 and 10 a.m., following an overnight fast. Women, as previously documented [38, 39], were attired in light clothing without shoes during the evaluation. Weight was assessed using a calibrated balance beam scale (Seca 711; Seca, Hamburg, Germany), and height was measured with a wall-mounted stadiometer (Seca 711; Seca, Hamburg, Germany). Subsequently, BMI was calculated as weight (kg) divided by height squared (m^2^).

In accordance with the World Health Organization (WHO)’s criteria, women were categorized as follows: a BMI of 25.0–29.9 kg/m^2^ indicated overweight, and a BMI within the range of 30.0–34.9 kg/m^2^ denoted grade I obesity [40]. Waist circumference (WC) was determined following the guidelines of the National Center for Health Statistics. A non-stretchable measuring tape was used at the natural indentation or at a midway level between the lower edge of the rib cage and the iliac crest if no natural indentation was visible. The measurements were recorded to the nearest 0.1 cm.

### Body composition

Body composition was evaluated using a BIA phase-sensitive system administered by a certified clinical nutrition specialist with 5 years of expertise in employing the BIA method for body composition assessment (800-µA current at a single frequency of 50 kHz, BIA 101, RJL Akern Bioresearch, Florence, Italy) [41], in accordance with previously documented procedures [39, 42, 43]. The BIA analysis adhered to the guidelines set by the European Society of Parental and Enteral Nutrition (ESPEN) [44]. Women were instructed to remove their shoes and socks, and the electrode contact areas (BIATRODES Akern Srl; Florence, Italy) were cleansed with alcohol immediately before placement on the hand and the ipsilateral foot, following the protocol outlined by Kushner [45]. Phase angle (PhA) was computed using the relationship between resistance (R) and reactance (Xc) based on the formula: PhA (°, degrees) = Xc/R* (180/π).

The BIA data were acquired under strictly standardized conditions, with women refraining from drinking, eating, and exercising for 6 h and abstaining from alcohol consumption within 24 h prior to testing. Women assumed a supine position with their limbs slightly separated from the body. The BIA examination was consistently conducted by the same nutritionist using the identical device to mitigate potential interobserver and interdevice variations. Regular checks of the BIA tool were performed with resistors and capacitors of known values, demonstrating reliability with within-day and between-day measurement variations of < 1.4% for R, < 1.5% for Xc, and < 1.7% for R, < 2.0% for Xc, respectively. The coefficient of variation (CV) for repeated measurements of R and Xc at 50 kHz was assessed in 8 individuals, yielding CVs of 1.3% for R and 1.2% for Xc.

### Laboratory parameters

Reactive oxygen metabolites (dROMs) were evaluated as biomarkers indicative of oxidative stress using an automated analyzer (Free Carpe Diem, Diacron International, Grosseto, Italy) and corresponding commercial kits (Diacron International) [46, 47]. Specifically, for dROMs assessment, 10 µL of serum was transferred into 1 cm cuvettes containing 1 mL of R2 reagent (acetate buffer, pH 4.8). The resulting mixture was gently mixed, and 10 µL of R1 reagent (a chromogenic mixture comprising aromatic alkyl-amine, A-NH2) was added. After inversion mixing, the samples were read at 546 nm (5 min, 37 °C) using an automated analyzer.

dROMs, which are oxygen metabolites generated by free radical attacks at the expense of biomolecules, were stable and quantifiable. Specifically, the test employed here is based on Fenton's reaction, where, in the presence of iron, dROMs in serum generate alkoxyl (R − O*), and peroxyl (R − OO*) radicals. These radicals, in turn, oxidize an alkyl-substituted aromatic amine, producing a photometrically quantified pink-colored derivative ([A − NH2*]^+^) [48, 49]. dROMs are considered valuable biomarkers of oxidative stress, with determined ranges as follows: (i) normal: 250–300 Units Carratelli (UCARR), (ii) borderline: 300–320 UCARR, (iii) mild oxidative stress: 321–340 UCARR, (iv) moderate oxidative stress: 341–400 UCARR, (v) high oxidative stress: 401–500 UCARR, and (vi) very high oxidative stress: > 500 UCARR, where 1 UCARR = 0.08 mg H_2_O_2_/dL [48, 49].

The reliability of the analysis was assessed by calculating the CV % at both intra- and inter-assay levels for all collected samples, resulting in an estimated CV % below 2.72% for both parameters.

### Determination of circulating levels of TMAO

Serum levels of TMAO were measured in samples stored at − 80 °C, a condition demonstrated to maintain TMAO stability for several years in a previous study [50]. The quantification of circulating TMAO levels followed the method outlined by Beale and Airs [50], as detailed in our prior research [51, 52], with minor adjustments. In summary, serum proteins were precipitated using methanol (serum:methanol, 1:2, v/v); the samples were vortex-mixed for 2 min, centrifuged at 14,000*g* for 10 min (4 °C), and the supernatants were collected and subjected to analysis using the High-Performance Liquid Chromatography-Mass Spectrometry (HPLC–MS) method [53]. The HPLC–MS conditions and method optimization adhered to Beale and Airs [54]. The HPLC system Jasco Extrema LC-4000 system (Jasco Inc., Easton, MD, USA) was coupled to a single quadrupole mass spectrometer (Advion ExpressIonL CMS, Advion Inc., Ithaca, NY, USA) equipped with an electrospray ionization (ESI) source, operating in positive ion mode. Chromatographic separation utilized a Luna hydrophilic interaction liquid chromatography (HILIC) column (150 × 3 mm, 5 µm particles) along with a guard column, both provided by Phenomenex (Torrance, CA, USA).

The sensitivity of the analytical method was described by the determination of Limit of Detection (LoD) of 2 ng/mL and Limit of Quantification (LoQ) of 6 ng/mL. In order to evaluate the precision of the method used, the CV% at intra- and inter-day level was calculated at three different TMAO levels (0.3, 3, and 13 µM), resulting in a calculated intra-day CV% of 8.12, 1.54, and 1.52 µM and of inter-day CV% of 9.2, 2.2, and 3.3 µM, respectively. Similarly, over the same TMAO levels, the accuracy of the method was calculated by the evaluation of the accuracy (% bias) both intraday and interday, leading to an estimation of % bias ranging from—3.52 to 0.66, indicating of high reliability of the used LC/MS method.

### VLCKD intervention

According to EASO guidelines [34] and the consensus statement from the working group of the Club of the Italian Society of Endocrinology (SIE)-diet therapies in endocrinology and metabolism [55], VLCKD consists of different phases (active—ketogenic—, re-education—non-ketogenic—, and maintenance). This study evaluated only the active phase, the ketogenic one.

The dietary composition adhered to specific parameters, with a total energy intake of less than 800 kcal *per* day. This energy was derived from a distribution of 13% from carbohydrates (less than 30 g *per* day), 43% from protein (1.3 g *per* kilogram of ideal body weight), and 44% from fat. The ideal body weight (kg) was calculated using the Lorentz equation: ideal body weight = height (cm) − 100 − [(height − 150)/2] [56]. Throughout VLCKD, meals with high biological value were provided as replacements, and the protein content originated from sources such as whey, soy, eggs, and peas. To ensure nutritional adequacy during the VLCKD, supplementation was introduced. This included B-complex vitamins, vitamins C and E, essential minerals like potassium, sodium, magnesium, and calcium, as well as omega-3 fatty acids. The active phase of VLCKD was collaboratively devised by a Nutritionist and endorsed by an Endocrinologist. The schematic representation of the active phase of VLCKD according to KeNuT multisteps dietary protocol with meal replacements proposed by the Club of the Italian Society of Endocrinology (SIE)—Diet Therapies in Endocrinology and Metabolism are reported in Table S1 (Additional file 1).

### Statistical analysis

The MedCalc® package (Version 12.3.0, 1993–2012 MedCalc Software bvba—MedCalc Software, Mariakerke, Belgium) and IBM SPSS Statistics Software (PASW Version 21.0, SPSS Inc., Chicago, IL, USA) were employed for the data analysis. The statistical analysis specifically focused on women with measurements at both baseline and after 45 days of the active phase of VLCKD. Results were expressed as mean ± standard deviation (SD) for continuous variables and as a number and percentage (n, %) for categorical variables. The Kolmogorov–Smirnov test was used to assess data distribution, and the paired Student’s *t*-test was utilized to compare differences between baseline and measurements after 45 days of the active phase of VLCKD. Spearman’s correlation was applied to assess the association between baseline and measurements after 45 days of the VLCKD phase in terms of percentage changes (∆%).

## Results

The study population included 31 women with treatment-naïve moderate acne (19 ≥ GAGS ≤ 30, median value 24), grade I obesity (BMI 30.03 to 34.65 kg/m^2^, median value 33.05 kg/m^2^), aged 18 to 30 years.

Anthropometric characteristics and body composition of the study population at baseline and after 45 days of the active phase of VLCKD are reported in Table 1. After 45 days of the active phase of VLCKD, in the entire study population, both BMI (Δ%: − 8.08 ± 1.52, *p* < 0.001) and WC (Δ%: − 7.51 ± 1.67, *p* < 0.001) were significantly reduced compared to baseline. After 45 days of the active phase of VLCKD, fat mass (FM) (kg and %) (Δ%: − 11.34 ± 4.90 and − 11.34 ± 4.90, both *p* < 0.001) and fat free mass (FFM) (kg) (Δ%: − 1.66 ± 1.38, *p* < 0.001) were significantly reduced while FFM (%) (Δ%: + 7.02 ± 2.32, *p* < 0.001) slightly increased. A significant increase in PhA (Δ%: + 8.60 ± 7.40, *p* < 0.001) compared to the baseline was also detected.

**Table 1:** Baseline and post-45-day VLCKD anthropometric and body composition parameters of women with acne and obesity

Parameters (N = 31)	Baseline	Post 45 days of active phase of VLCKD	*p*-value	∆%
Anthropometric parameters				
Weight (kg)	88.90 ± 6.12	81.73 ± 5.93	**<** **0.001**	− 8.07 ± 1.52
BMI (kg/m^2^)	32.69 ± 1.34	30.05 ± 1.37	**<** **0.001**	− 8.08 ± 1.52
Overweight (n, %)	–	12 (38.7)	χ^2^ = 12.50, ***p*** **<** **0.001**	
Grade I obesity (n, %)	31 (100.0)	19 (61.3)		
WC (cm)	97.81 ± 4.46	90.43 ± 4.98	**<** **0.001**	− 7.51 ± 1.67
< cut off *	–	11 (35.5)	χ^2^ = 11.50, ***p*** **<** **0.001**	
> cut off *	31 (100.0)	20 (64.5)		
Body composition parameters				
R (Ω)	480.68 ± 55.01	484.58 ± 53.14	**<** **0.001**	0.91 ± 2.88
Xc (Ω)	48.45 ± 6.44	52.87 ± 6.14	**<** **0.001**	9.69 ± 8.89
PhA (°)	5.75 ± 0.34	6.24 ± 0.41	**<** **0.001**	8.60 ± 7.40
TBW (lt)	40.08 ± 3.23	39.15 ± 3.00	**<** **0.001**	− 2.26 ± 1.60
TBW (%)	45.18 ± 3.69	48.04 ± 3.91	**<** **0.001**	6.35 ± 2.07
ECW (lt)	18.80 ± 1.68	17.48 ± 1.47	**<** **0.001**	− 6.87 ± 4.47
ECW (%)	46.90 ± 1.61	44.70 ± 1.78	**<** **0.001**	− 4.66 ± 3.79
ICW (lt)	21.27 ± 1.80	21.67 ± 1.85	**0.003**	1.91 ± 3.26
ICW (%)	53.10 ± 1.61	55.31 ± 1.78	**<** **0.001**	4.22 ± 3.62
FM (kg)	34.78 ± 5.45	28.50 ± 5.50	**<** **0.001**	− 18.43 ± 5.48
FM (%)	38.95 ± 4.36	34.66 ± 5.10	**<** **0.001**	− 11.34 ± 4.90
FFM (kg)	54.14 ± 3.83	53.24 ± 3.81	**<** **0.001**	− 1.66 ± 1.38
FFM (%)	61.05 ± 4.36	65.34 ± 5.10	**<** **0.001**	7.02 ± 2.32
BCM (kg)	28.40 ± 2.23	29.21 ± 2.45	**0.001**	2.91 ± 4.20
BCMI (kg/m^2^)	10.45 ± 0.76	10.76 ± 0.87	**<** **0.001**	3.05 ± 4.15
SMM (kg)	226.42 ± 3.12	26.21 ± 2.92	0.101	− 0.68 ± 2.47
SMM (%)	29.82 ± 3.67	32.18 ± 3.89	**<** **0.001**	8.00 ± 3.06

*VLCKD* very low-calorie ketogenic diet, *BMI* body mass index, *WC* waist circumference, *R* resistance, *Xc* reactance, *PhA* phase angle, *TBW* total body water, *ECW* extracellular water, *ICW* intracellular water, *FM* fat mass, *FFM* fat free mass, *BCM* body cell mass, *BCMI* body cell mass index, *SMM* skeletal muscle mass

A p-value in bold type denotes a significant difference (p < 0.05)

^*^ WC 88 cm and 102 cm for females and males, respectively

Parameters of dysbiosis (TMAO) and oxidative stress (dROMs) in the study population at baseline and after 45 days of the active phase of VLCKD are reported in Table 2. After 45 days of the active phase of VLCKD, in the entire study population, we observed significant reductions in TMAO (Δ%: − 51.97 ± 15.98, *p* < 0.001) and dROMs (Δ%: − 38.07 ± 18.40, *p* < 0.001) levels compared to baseline.

**Table 2:** Baseline and post-45-day VLCKD dysbiosis and oxidative stress parameters of women with acne and obesity

Parameters (N = 31)	Baseline	Post 45 days of active phase of VLCKD	*p*-value	∆%
TMAO (µM)	5.20 ± 1.70	2.60 ± 1.39	**<** **0.001**	− 51.97 ± 15.98
dROMS (U Carr)	369.65 ± 50.78	224.64 ± 58.89	**<** **0.001**	− 38.07 ± 18.40
Normal (250–300 U Carr) (n, %)	–	28 (90.3)	χ^2^ = 47.48, ***p*** **<** **0.001**	
Borderline (300–320 U Carr) (n, %)	2 (6.5)	1 (3.2)	χ^2^ = 0.01, *p* = 0.999	
Mild oxidative stress (321–340 U Carr) (n, %)	8 (25.8)	2 (6.5)	χ^2^ = 2.98, *p* = 0.084	
Moderate oxidative stress (341–400 U Carr) (n, %)	14 (45.2)	–	χ^2^ = 15.59, ***p*** **<** **0.001**	
High oxidative stress (401–500 U Carr) (n, %)	7 (22.6)	–	χ^2^ = 5.80, ***p*** **=** **0.016**	

*VLCKD* very low-calorie ketogenic diet, *Δ%* percentage change, *dROMS* reactive oxygen metabolites, *TMAO* trimethylamine *n*-oxide

A p-value in bold type denotes a significant difference (p < 0.05)

**Fig. 3:**
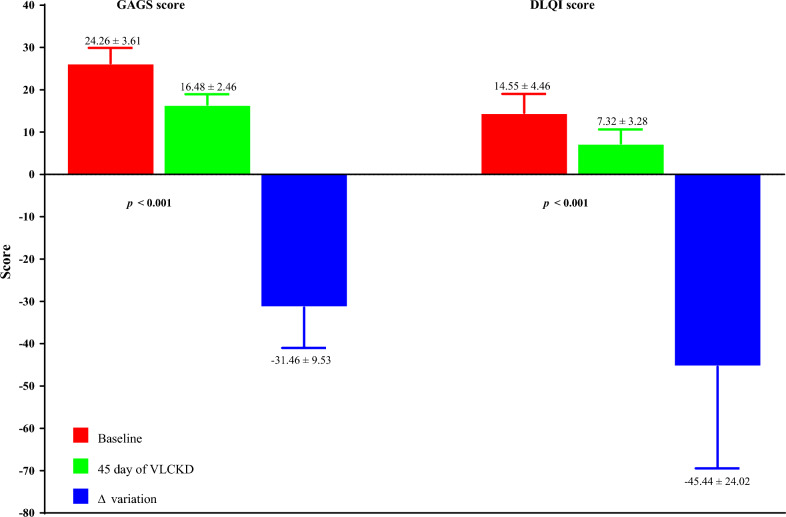
Baseline and post-45-day VLCKD dermatological parameters of women with acne. A p-value in bold type denotes a significant difference (p < 0.05). *VLCKD* very low-calorie ketogenic diet, *Δ%* percentage change, *GAGS* global acne grading system, *DLQI* dermatology life quality index

The dermatological parameters of the study population at baseline and after 45 days of the active phase of VLCKD are shown in Table 3. Of note, after 45 days of the active phase of VLCKD, both the GAGS score (Δ%: − 31.46 ± 9.53, *p* < 0.001), and the DLQI score (Δ%: − 45.44 ± 24.02, *p* < 0.001) decreased significantly compared to baseline (Fig. 3).

**Table 3:** Baseline and post-45-day VLCKD dermatological parameters of women with acne and obesity

Parameters (N = 31)	Baseline	Post 45 days of active phase of VLCKD	*p*-value	∆%
GAGS	24.26 ± 3.61	16.48 ± 2.46	**<** **0.001**	− 31.46 ± 9.53
Mild-severity (1–18)	–	27 (87.1)	χ^2^ = 44.35, ***p*** **<** **0.001**	
Moderate-severity (19–30)	31 (100.0)	4 (12.9)		
DLQI	14.55 ± 4.46	7.32 ± 3.28	**<** **0.001**	− 45.44 ± 24.02
Small effect on QoL (2–5)	1 (3.2)	10 (32.3)	χ^2^ = 7.07, ***p*** **=** **0.008**	
Moderate effect on QoL (6–10)	5 (16.1)	16 (51.6)	χ^2^ = 7.20, ***p*** **=** **0.007**	
Very large effect on QoL (11–20)	22 (71.0)	5 (16.1)	χ^2^ = 16.80, ***p*** **<** **0.001**	
Extremely large effect on QoL (21–30)	3 (9.7)	–	χ^2^ = 1.40, *p* = 0.237	

*VLCKD* very low-calorie ketogenic diet, *Δ%* percentage change, *GAGS* global acne grading system, *DLQI* dermatology life quality index, *QoL* quality of life

A p-value in bold type denotes a significant difference (p < 0.05)

**Fig. 4:**
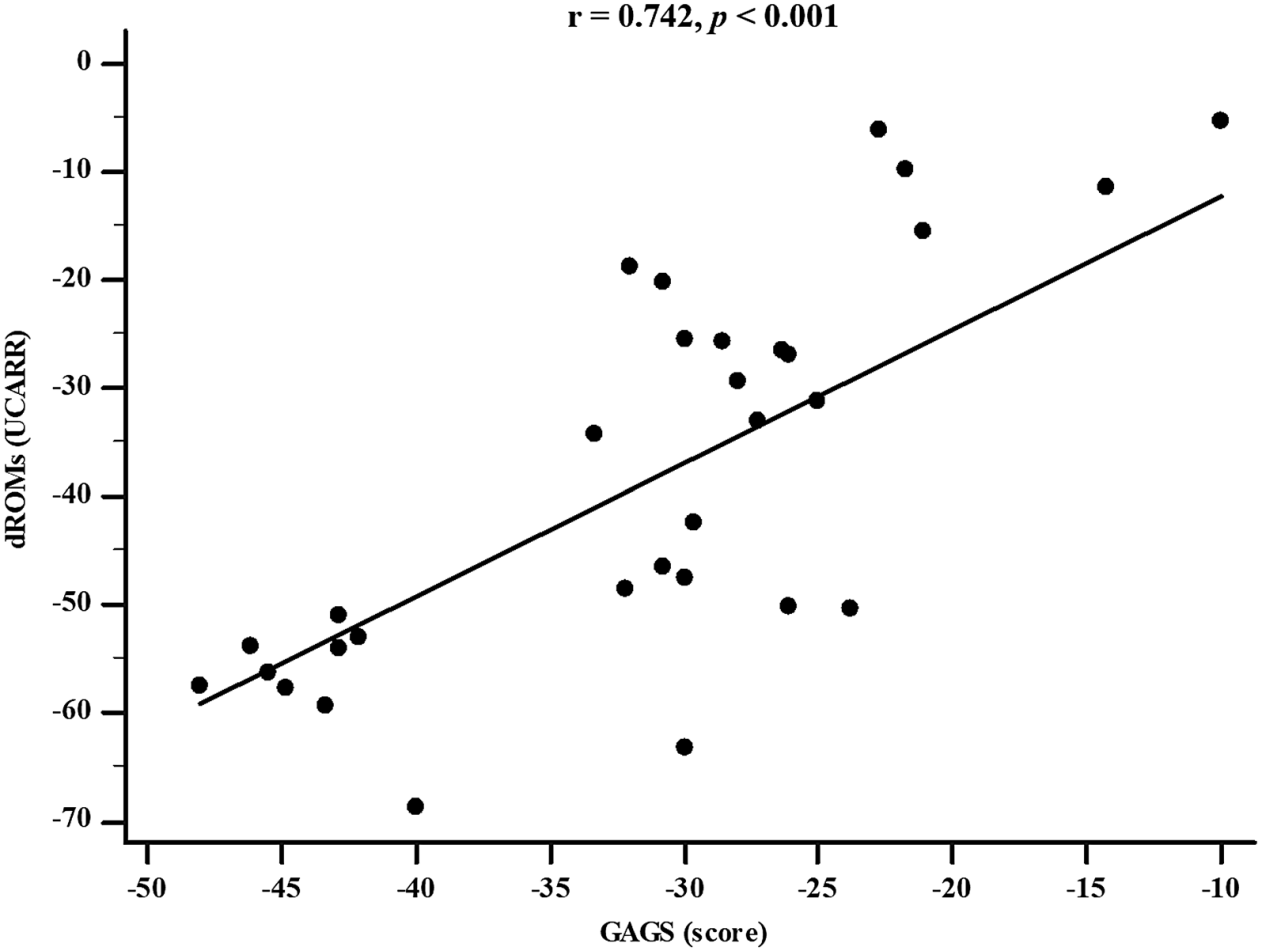
Correlation between GAGS score and dROMs levels after 45 days of active phase of VLCKD (adjusted for Δ% FM). *GAGS* global acne grading system, *dROMs* reactive oxygen metabolites

Table 4 reports the simple and adjusted correlations among changes in the GAGS score and changes in the study parameters after 45 days of the active phase of VLCKD. Changes in the GAGS score positively correlated with changes in weight (*p* = 0.004), BMI (*p* = 0.001), WC (*p* = 0.012), total body water (TBW) (lt) (*p* = 0.001), extracellular water (ECW) (lt) (*p* < 0.001), ECW (%) (*p* = 0.001), FM (kg) (*p* = 0.023), skeletal muscle mass (SMM) (*p* = 0.012), TMAO (*p* = 0.020), dROMs (*p* < 0.001) and DLQI (*p* < 0.001) and negatively with R (*p* = 0.010), Xc (*p* < 0.001), PhA (*p* < 0.001), ICW (%) (*p* = 0.001), BCM (*p* = 0.017), and BCMI (*p* = 0.016). Interestingly, the correlations with dROMs (*p* < 0.001) (Fig. 4) and PhA (*p* = 0.005) were maintained even after adjustment for Δ% FM.

**Table 4:** Simple and adjusted correlations among the Δ% GAGS score and changes in the study parameters after 45 days of the active phase of VLCKD

Δ%	Δ% GAGS			
	Simple		Adjusted for Δ% FM	
	*r*	p-value	*r*	p-value
Anthropometric parameters				
Weight (kg)	0.505	**0.004**	0.327	0.078
BMI (kg/m^2^)	0.507	**0.004**	0.332	0.073
WC (cm)	0.445	**0.012**	0.240	0.201
Body composition parameters				
R (Ω)	− 0.456	**0.010**	− 0.550	**0.002**
Xc (Ω)	− 0.657	**<** **0.001**	− 0.565	**0.001**
PhA (°)	− 0.603	**<** **0.001**	− 0.502	**0.005**
TBW (lt)	0.547	**0.001**	0.570	**0.001**
TBW (%)	0.031	0.868	0.417	**0.022**
ECW (lt)	0.660	**<** **0.001**	0.574	**0.001**
ECW (%)	0.583	**0.001**	0.470	**0.009**
ICW (lt)	− 0.342	0.060	− 0.017	0.929
ICW (%)	− 0.582	**0.001**	− 0.462	**0.010**
FM (kg)	0.407	**0.023**	–	**–**
FM (%)	0.345	0.057	–	–
FFM (kg)	0.329	0.071	0.601	**<** **0.001**
FFM (%)	− 0.175	0.346	0.465	**0.010**
BCM (kg)	–0427	**0.017**	− 0.161	0.396
BCMI (kg/m^2^)	− 0.428	**0.016**	− 0.164	**0.387**
SMM (kg)	0.455	**0.012**	0.545	**0.002**
SMM (%)	0.115	0.540	0.456	**0.011**
Dysbiosis and oxidative stress parameters				
TMAO (µM)	0.415	**0.020**	0.130	0.495
dROMS (U Carr)	0.775	**<** **0.001**	0.756	**<** **0.001**
Dermatological parameters				
DLQI (score)	0.736	**<** **0.001**	0.683	**<** **0.001**

A p-value in bold type denotes a significant difference (p < 0.05)

*GAGS* global acne grading system, *DLQI* dermatology life quality index, *BMI* body mass index, *WC* waist circumference, *R* resistance, *Xc* reactance, *PhA* phase angle, *TBW* total body water, *ECW* extracellular water, *ICW* intracellular water, *FM* fat mass, *FFM* fat free mass, *BCM* body cell mass, *BCMI* body cell mass index, *SMM* skeletal muscle mass, *dROMS* reactive oxygen metabolites, *TMAO* trimethylamine *n*-oxide

Table 5 reports the simple and adjusted correlations among changes in the DLQI score and changes in the study parameters after 45 days of the active phase of VLCKD. Changes in the DLQI score positively correlated with changes in weight (*p* < 0.001), BMI (*p* < 0.001), WC (*p* = 0.002), ECW (lt) (*p* < 0.001), ECW (%) (*p* < 0.001), FM (kg) (*p* < 0.001), FM (%) (*p* < 0.001), TMAO (*p* < 0.001), dROMs (*p* < 0.001) and GAGS (*p* < 0.001) and negatively with Xc (*p* < 0.001), PhA (*p* < 0.001), ICW (lt) (*p* < 0.001), ICW (%) (*p* = 0.001), FFM (%) (*p* = 0.002), BCM (*p* < 0.001) and BCMI (*p* < 0.001). Of note, the correlations with dROMs (*p* < 0.001) and PhA (*p* < 0.001) were maintained even after adjustment for Δ% FM.

**Table 5:** Simple and adjusted correlations among the Δ% DLQI score and changes in the study parameters after 45 days of the active phase of VLCKD

Δ%	Δ% DLQI			
	Simple		Adjusted for Δ% FM	
	*r*	p-value	*r*	p-value
Anthropometric parameters				
Weight (kg)	0.719	**<** **0.001**	0.408	**0.025**
BMI (kg/m^2^)	0.721	**<** **0.001**	0.412	**0.024**
WC (cm)	0.533	**0.002**	0.102	0.593
Body composition parameters				
R (Ω)	− 0.188	0.312	− 0.351	0.057
Xc (Ω)	− 0.751	**<** **0.001**	− 0.573	**0.001**
PhA (°)	− 0.827	**<** **0.001**	− 0.656	**<** **0.001**
TBW (lt)	0.333	0.068	0.388	**0.034**
TBW (%)	− 0.334	0.066		
ECW (lt)	0.781	**<** **0.001**	0.593	**0.001**
ECW (%)	0.813	**<** **0.001**	0.625	**<** **0.001**
ICW (lt)	− 0.707	**<** **0.001**	− 0.377	**0.040**
ICW (%)	− 0.814	**<** **0.001**	− 0.631	**<** **0.001**
FM (kg)	0.667	**<** **0.001**	–	–
FM (%)	0.600	**<** **0.001**	–	–
FFM (kg)	0.028	0.879	0.453	**0.012**
FFM (%)	− 0.535	**0.002**	0.190	**0.315**
BCM (kg)	− 0.748	**<** **0.001**	− 0.453	**0.012**
BCMI (kg/m^2^)	− 0.747	**<** **0.001**	− 0.451	**0.012**
SMM (kg)	0.167	0.369	0.335	0.070
SMM (%)	− 0.262	0.154	0.185	0.326
Dysbiosis and oxidative stress parameters				
TMAO (µM)	0.597	**<** **0.001**	0.023	0.904
dROMS (U Carr)	0.914	**<** **0.001**	0.838	**<** **0.001**
Dermatological parameters				
GAGS (score)	0.736	**<** **0.001**	**0.683**	**<** **0.001**

A p-value in bold type denotes a significant difference (p < 0.05)

*GAGS* global acne grading system, *DLQI* dermatology life quality index, *BMI* body mass index, *WC* waist circumference, *R* resistance, *Xc* reactance, *PhA* phase angle, *TBW* total body water, *ECW* extracellular water, *ICW* intracellular water, *FM* fat mass, *FFM* fat free mass, *BCM* body cell mass, *BCMI* body cell mass index, *SMM* skeletal muscle mass, *dROMS* reactive oxygen metabolites, *TMAO* trimethylamine *n*-oxide

## Discussion

In this study, a cohort of 31 women with treatment-naïve moderate acne and grade I obesity underwent the active phase of VLCKD for 45 days. As expected, at the end of the active phase of VLCKD, anthropometric measurements showed significant reductions in both BMI and WC, and, for body composition, FM (kg and %) decreased significantly, while FFM (%) showed a slight increase. In addition, there were also notable increments observed in PhA, and this was consistent with previous research [39, 42, 43]. PhA is a BIA parameter that serves as an indicator of cellular health and the distribution of body fluids. It has been recognized as a prognostic marker for both the incidence of illnesses and the likelihood of mortality in cases of chronic inflammatory conditions [57]. It’s worth noting that PhA values tend to be diminished in a significant portion of inflammatory disorders, which encompass conditions like psoriasis and hidradenitis suppurativa [58, 59].

The results of this study are promising for acne patients. The results of our study represented a novel finding, as they showed a significant reduction in TMAO and dROMs levels after the 45-day active phase of VLCKD in women with acne and obesity. This novel finding not only underscored the potential efficacy of VLCKD in the management of these conditions but also indicated a positive impact on oxidative stress and gut dysbiosis, both potential mechanisms influencing acne severity. Together, in fact, the simultaneous decrease in the GAGS score and DLQI score added an additional level of significance to our results. This dual improvement not only suggests an improvement in acne severity but also highlights a substantial improvement in participants’ overall quality of life. These findings carry significant implications regarding the potential benefits of VLCKD, particularly for patients struggling with acne and obesity, a category of patients particularly exposed to dysbiosis, oxidative stress, and a high risk of cardiovascular diseases [60–62].

In this scenario, the decline in oxidative stress, as indicated by reduced levels of dROMs, and the amelioration of gut dysbiosis, represented by decreased TMAO levels, induced by VLCKD, may collectively constitute the underlying pathophysiological mechanism associated with the beneficial outcomes of this dietary therapy in mitigating the clinical severity of acne. Presently, there exists a substantial gap in the scientific literature regarding the utilization of VLCKDs in diverse skin disorders [63, 64]. Despite some evidence supporting the use of VLCKDs in psoriasis [32], there is a notable absence of clinical studies, to the best of our knowledge, assessing the effectiveness of VLCKD in treating acne.

Interestingly, gut dysbiosis is implicated in elevating systemic inflammation, which correlates with the onset and clinical severity of acne [65]. Moreover, studies indicate that regular intake of probiotics, particularly those containing lactobacillus strains, over 12 weeks is associated with a significant reduction in inflammatory acne lesions (30% to 67%) and a concurrent decrease in IGF-1 levels by 32% [66–69].

Although limited research has explored the connection between VLCKDs and microbiota, both human and animal studies report positive effects on restructuring bacterial composition and enhancing gut biological function, fostering an increase in anti-inflammatory bacteria [70]. VLCKDs may influence the gut microbiota through metabolites produced by various bacteria, resulting in improved short-chain fatty acids production, reduced lactate, and increased hydrogen sulfide [70].

Recent evidence suggests that elevated insulin levels may contribute significantly to acne development through effects on sex hormones, subsequently influencing sebum production and inflammation. VLCKDs are associated with reduced insulin levels, leading to a decline in IGF-1 levels [71], ultimately triggering an increase in IGFBP-3 levels [64, 72]. This reduction in insulin and IGF-1 levels contributes to heightened SHBG levels, leading to decreased androgen production and circulation, even in the skin, correlating with diminished sebum production [63, 64, 72]. The decline in IGF-1 levels, induced by VLCKDs, may attenuate IGF-1 signaling, leading to decreased androgen synthesis and inhibition of the AKT-mTORC1 pathway [73, 74]. The subsequent reduction in androgen levels via mTORC2-mediated AKT inactivation [75, 76], along with the increased expression of Domain Containing MTOR Interacting Protein (DEPTOR), an inhibitor of mTORC1 and mTORC2 negatively regulated by androgen receptor, further enhances mTORC1 inhibition [77].

The reduction in sebum quantity and quality hampers the overgrowth of *Propionibacterium acnes*, thereby improving the skin biofilm [76]. Lower levels of *Propionibacterium acnes*-derived lipoteichoic acid and free palmitic acid act via TLR2 to inhibit the activation of the NLRP3 inflammasome, thereby reducing Th17 cell-driven inflammation and inhibiting pro-inflammatory cytokine secretion, including IL-1β and IL-1 release [76].

Moreover, the anti-inflammatory properties of ketone bodies, extensively discussed in a recent review [78], are likely beneficial for the inflammatory nature of acne, leading to a reduction in both systemic and local inflammatory processes.

Given these factors, including the improvement of the gut microbiota and the reduction of inflammation and oxidative stress, it is hypothesized that VLCKDs may contribute to diminishing the development and clinical severity of acne.

Limitations of the study were:

- The sample was limited, and this may have affected the generalizability of the results. However, we used accurate inclusion and exclusion criteria to increase the value of any results;
- Lack of a control group; however, for the short treatment period, a comparison with another diet, such as the Mediterranean diet, would have been ineffective, requiring a longer period for comparable results;
- The sample included only women, limiting consideration of the effect of VLCKD on men. However, acne afflicts women more [6, 7] and our results could be better applied based on this sex disproportion.
- We did not evaluate C-reactive protein, which is often used as a marker of systemic inflammation, and this may affect the completeness of our evaluations. However, it is important to note that some studies suggest that C-reactive protein may not be an ideal marker in acne, as inflammation in this dermatologic condition is considered more localized than systemic [79, 80];
- A comprehensive analysis of safety and interactions with drugs used to treat acne has not been conducted; in fact, we only recruited treatment-naïve patients. Assessing these interactions will be crucial to ensuring the safety of the treatment;
- Further studies, preferably randomized, are needed to compare the efficacy and safety of VLCKD with other dietary therapies available for patients with acne;
- Another limitation of this study is that our results refer to a relatively short period of time. It would be interesting to know if these improvements are maintained over time.

We also outline the strengths of the study:

- The absence of dropouts is surely a strength of this study;
- VLCKD used highly controlled replacement meals, ensuring a strictly monitored caloric and nutritional intake. This contributed to maintaining a highly controlled and standardized diet for all participants;
- The patients were followed by a specialized multidisciplinary team that continuously monitored adherence to VLCKD. In detail, we constantly monitored levels of physical activity and diet adherence during the phone calls and follow-up visits;
- Stringent inclusion criteria were used. In detail, we applied very stringent inclusion criteria, including only young women of childbearing age, only with treatment-naïve (absence of any treatment for acne) moderate acne, grade I obesity, non-smokers, and those who do not regularly practice physical activity.

The practical implications of incorporating VLCKD into the clinical management of acne disease highlight its potential as a safe, cost-effective, and complementary treatment option, particularly for patients with obesity. This expands the range of treatment options available for individuals struggling with both acne and obesity, offering a potential solution that addresses both conditions simultaneously. However, its successful implementation requires careful consideration of individual patient characteristics, ongoing monitoring, and collaboration among healthcare professionals to ensure safety, efficacy, and long-term sustainability.

## Conclusion

With this study, we propose for the first time VLCKD as a possible therapeutic tool for young women with moderate acne and obesity. The results of this study are promising for acne patients. In this context, given the possible side effects of medications used for acne, there is a growing need for safe, tolerable, and low-cost alternative treatments that can be used to reduce the clinical severity of moderate acne in patients with obesity, possibly also as an adjunct to pharmacological therapy for acne disease, since it has been widely demonstrated that VLCKD is tolerable, safe, and effective. Therefore, VLCKD could be used in the repertoire of clinical management of acne disease within a multidisciplinary team that includes the presence of a qualified nutritionist.

### Supplementary Information

**Additional file 1: Table S1.** Schematic representation of the active phase of VLCKD according to KeNuT multisteps dietary protocol with meal replacements proposed by the Club of the Italian Society of Endocrinology (SIE)—Diet Therapies in Endocrinology and Metabolism.

## Data Availability

The datasets used and/or analyzed during the current study are available from the corresponding author on reasonable request.

## Abbreviations

QoL: Quality of life
DLQI: Dermatology Life Quality Index
PPARγ: Peroxisome proliferator-activated receptor γ
mTOR: Mammalian target of rapamycin
ROS: Reactive oxygen species
TLR: Toll-like receptor
TNFα: Tumor necrosis factor α
IL: Interleukin
IGF-1: Insulin-like growth factor 1
VLCKD: Very low calorie ketogenic diet
GAGS: Global Acne Grading Score
BMI: Body mass index
EASO: European Association for the Study of Obesity
BIA: Bioelectrical impedance analysis
TMAO: Trimethylamine *N*-oxide
WC: Waist circumference
ESPEN: European Society of Parental and Enteral Nutrition
PhA: Phase angle
R: Resistance
Xc: Reactance
CV: Coefficient of variation
dROMs: Reactive oxygen metabolites
UCARR: Units Carratelli
HPLC–MS: High-performance liquid chromatography-mass spectrometry
HILIC: Hydrophilic interaction liquid chromatography
ESI: Electrospray ionization
LoD: Limit of detention
LoQ: Limit of quantification
SD: Standard deviation
FM: Fat mass
FMM: Fat free mass
TBW: Total body water
ECW: Extracellular water
SMM: Skeletal muscle mass
DEPTOS: Domain containing MTOR interacting protein
