# Supplementary material for: Very low-calorie ketogenic diet (VLCKD): a therapeutic nutritional tool for acne?
Source: J Transl Med. 2024 Mar 31;22:322. doi: 10.1186/s12967-024-05119-5 (PMC10983624; doi:10.1186/s12967-024-05119-5)
Supplement: Supplementary file 1 — Additional file 1: Table S1. Schematic representation of the active phase of VLCKD according to KeNuT multisteps dietary protocol with meal replacements proposed by the Club of the Italian Society of Endocrinology (SIE)—Diet Therapies in Endocrinology and Metabolism. [file 12967_2024_5119_MOESM1_ESM.docx]

| Meal | Consumption of | Addendum |
| --- | --- | --- |
| Breakfast | high-biological-value protein meal replacement |  |
| Snack | high-biological-value protein meal replacement |  |
| Lunch | high-biological-value protein meal replacement | low glycemic index/load and low-sugar vegetables + 1 tablespoon of extra virgin olive oil |
| Dinner | high-biological-value protein meal replacement | low glycemic index/load and low-sugar vegetables + 1 tablespoon of extra virgin olive oil |
| Mandatory supplementation of micronutrients (vitamins, such as complex B vitamins, vitamin C and E, minerals, including potassium, sodium, magnesium, calcium; and omega-3 fatty acids) | | |

**Table S1.** Schematic representation of the active phase of VLCKD according to KeNuT multisteps dietary protocol with meal replacements proposed by the Club of the Italian Society of Endocrinology (SIE)—Diet Therapies in Endocrinology and Metabolism.
